# Fluorescence spectroscopy precorrection model robust to large variability of optical properties: application to point of care quantitative liver graft viability assessment

**DOI:** 10.1364/BOE.587424

**Published:** 2026-04-13

**Authors:** Antoine Uzel, Arthur Gautheron, Olivier Lopez, Guillaume Rossignol, Xavier Muller, Natacha Boulanger, Michaël Sdika, Bruno Montcel

**Affiliations:** 1 INSA-Lyon, Universite Claude Bernard Lyon 1, CNRS, Inserm, CREATIS UMR5220, U1294, F-69100, Lyon, France; 2 CPE Lyon, Lyon, France; 3 Aix Marseille Université, LIIE, Marseille, France; 4 Aix Marseille Université, CERIMED, Marseille, France; 5 Department of Pediatric Surgery, Hôpital Femme Mère Enfant, Hospices Civils de Lyon, University of Lyon I, 69500 Lyon, France; 6 Department of Surgery and Liver Transplantation, Croix-Rousse University Hospital, University of Lyon I, Lyon, France; 7 Institut d’Hépatologie de Lyon, UMR INSERM U1350 PaThLiv, IHU EVEREST, Lyon, France; 8 antoine.uzel@creatis.insa-lyon.fr; 9 bruno.montcel@creatis.insa-lyon.fr

## Abstract

Optical properties correction models for fluorescence spectroscopy faced limitations due to the high variability of optical properties. Typically, the point-of-care assessment of liver graft viability remains a critical challenge in transplantation medicine, where current methods rely on subjective visual inspection and delayed laboratory biomarkers. While optical approaches offer promising solutions to this challenge, the unique characteristics of liver grafts, which undergo highly varying conditions from procurement to transplantation, ranging from blood perfusion to ischemic phases and perfusion with transparent solutions, create an extremely wide dynamic range of optical properties that challenge state-of-the-art optical models. We present a novel method combining fluorescence spectroscopy and diffuse reflectance measurements, accounting for the large variability in optical properties and adapted for liver graft viability assessment. Our approach corrects for tissue optical properties using a dual-modality system that measures both fluorescence and diffuse reflectance spectra, enabling accurate quantification of metabolic fluorophores such as NADH and FAD. The method was validated on tissue-mimicking phantoms with varying optical properties and demonstrated superior performance compared to state-of-the-art correction techniques, with quantification accuracy improved by at least a factor of 1.5 and a capacity to detect more than 92% of the fluorophores present in the different phantoms. Clinical validation was performed on porcine ischemia-reperfusion models, showing promising results for real-time tissue viability monitoring during organ preservation and transplantation procedures.

## 1. Introduction

Endogenous fluorescence spectroscopy has emerged as a powerful tool for non-invasive tissue characterization, offering unique insights into cellular and molecular processes without the need for exogenous contrast agents or invasive sampling procedures. Endogenous tissue fluorescence arises from numerous naturally occurring molecules [1], each providing specific biological information. Protoporphyrin IX (PpIX) has proven valuable for brain tumor detection and delineation during fluorescence-guided surgery [2,3]. Collagen and elastin provide information about extracellular matrix composition and tissue structural integrity [4–6]. Among these endogenous fluorophores, Nicotinamide Adenine Dinucleotide (NADH) and Flavin Adenine Dinucleotide (FAD) are particularly significant as they serve as direct reporters of cellular energy metabolism [7–9]. These coenzymes play central roles in cellular respiration, with NADH primarily associated with glycolysis and the citric acid cycle, while FAD participates in oxidative phosphorylation. The redox ratio, typically defined as [FAD]/([FAD]+[NADH]), offers a quantitative measure of the balance between oxidative phosphorylation and glycolysis, enabling assessment of cellular metabolic state and tissue viability [10].

However, accurate quantification of tissue fluorescence remains challenging due to the confounding effects of tissue optical properties on measured fluorescence signals. The presence of chromophores, particularly blood with its strong hemoglobin absorption, introduces attenuation in the spectral regions where many fluorophores have their excitation and emission maxima in UV-visible wavelength. This absorption not only attenuates the excitation light reaching the fluorophores but also affects the collection of emitted fluorescence, resulting in distorted measurements that do not accurately reflect the true fluorophore concentrations and emission spectra [11–13]. Tissue scattering, mainly caused by cell nuclei, further complicates the relationship between measured fluorescence and actual fluorophore content by modifying both excitation light distribution and fluorescence collection efficiency. Without proper correction for these optical effects, fluorescence measurements can be misinterpreted, leading to erroneous conclusions about tissue metabolic state. To address these challenges, numerous attenuation correction techniques have been developed. Empirical correction methods typically rely on measuring the ratio of fluorescence to diffuse reflectance signals, attempting to compensate for tissue optical properties through specific wavelength relationships. Theory based techniques combine fluorescence measurements with diffuse reflectance spectroscopy to extract tissue optical properties and subsequently correct fluorescence signals using analytical models derived from radiative transport theory or diffusion approximations [14,15]. Monte Carlo simulation-based correction techniques utilize detailed numerical simulations of light propagation through tissue to model the relationship between intrinsic fluorescence and measured signals [16,17]. These correction approaches have demonstrated success in different specific clinical applications. For instance, Valdés et al. developed a spectrally constrained dual-band normalization technique for accurate PpIX quantification during fluorescence-guided surgery [18], enabling reliable tumor margin detection despite variations in tissue optical properties. Similarly, various correction methods have been successfully applied to quantify endogenous fluorophores like NADH and collagen in tissue characterization studies [19]. However, these correction techniques have typically been validated and applied in scenarios involving a limited number of fluorophores and relatively stable tissue optical properties within each measurement session. The success of these methods often relies on either fixed correction parameters calibrated for specific tissue types or reference measurements that assume consistent optical conditions across samples.

One clinical application that presents challenges beyond these operational constraints is organ transplantation, where the ability to monitor tissue metabolic status through fluorescence spectroscopy could address unmet needs in viability assessment. Organ transplantation faces a severe shortage, with demand exceeding the available donor pool, leading to increased numbers of patients dying while awaiting transplantation [20,21]. To address this shortage, the transplantation community has increasingly turned to extended criteria donors (ECD) and marginal grafts that would have been previously declined. Extended criteria donors include older donors (typically >60–65 years), donors with comorbidities such as diabetes, hypertension, or viral infections, and importantly, donation after circulatory death (DCD) cases where organs undergo additional warm ischemia time before procurement compared to traditional donation after brain death (DBD) [22–24]. DCD organs are particularly challenging as they experience prolonged ischemia-reperfusion injury, making real-time viability assessment crucial for transplant success [25]. However, current organ assessment methods rely primarily on visual inspection and histological examination, both subjective approaches that provide limited information about the metabolic state and functional viability of the organ [26]. These conventional methods are particularly inadequate for ECD organs, where the distinction between viable and non-viable tissue becomes critical for transplant decision-making. The ability to objectively assess organ viability could therefore expand the usable donor pool by enabling confident acceptance of extended criteria organs that are currently declined due to uncertain viability, while simultaneously reducing the risk of transplanting non-viable grafts. This shortage is particularly acute in liver transplantation, where increasing utilization of extended criteria donors has driven the development of machine perfusion technologies as promising solutions for organ preservation and assessment. Machine perfusion systems, including hypothermic oxygenated perfusion with preservation solution (HOPE) and normothermic machine perfusion (NMP), often with blood, maintain organs at different temperatures while providing oxygenated perfusion [27–31].

Fluorescence spectroscopy is particularly well-suited for continuous organ viability assessment during these complex perfusion protocols. Real-time monitoring of NADH and FAD fluorescence throughout the transplantation process, from procurement through preservation to implantation, could provide direct insight into cellular metabolic state and tissue viability. This metabolic monitoring capability would enable detection of metabolic recovery during reperfusion, guide preservation strategy optimization, and provide key information for transplantability decisions. While recent studies have demonstrated the potential of perfusate fluorescence analysis for liver assessment during HOPE [32–34], no comprehensive evaluation has been performed throughout a complete liver transplantation protocol combining multiple perfusion modalities. Moreover, current perfusion technologies provide limited direct information about tissue metabolic state during the preservation process [19,35–37]. However, realizing the full potential of fluorescence-based metabolic monitoring in this context requires correction methods capable of accurately quantifying multiple fluorophores (NADH, FAD). Indeed, during these perfusion protocols, organs experience variations in their optical environment as they transition between different perfusion phases with blood (high hemoglobin absorption), transparent preservation solutions (minimal absorption), or ischemic phases with no perfusion. These transitions, combined with temperature differences between hypothermic and normothermic conditions, create an environment where tissue absorption varies highly within hours. However, existing correction approaches, validated for scenarios with limited fluorophores and stable optical properties, are inadequate for this application where both the number of biomarkers and the variability of tissue optical properties far exceed their operational constraints.

To overcome these limitations, we present an improved correction method for fluorescence spectroscopy that builds upon existing approaches while addressing their adaptability constraints, specifically targeting applications in machine perfusion monitoring. To validate our correction methodology, we employ tissue-mimicking phantoms containing Acid Red 1 and Acid Red 14 dyes whose absorption spectra closely resemble those of oxygenated and deoxygenated hemoglobin. We added NADH and FAD as fluorophores and titanium dioxide nanoparticles (TiO_2_) as scattering agent. Using these blood-equivalent absorbers along with controlled fluorophore concentrations, we demonstrate that our method can accurately recover both fluorophore concentrations and absorber concentrations despite strong variations in optical properties, thereby validating both the fluorescence correction algorithm and the capability to measure tissue oxygenation parameters. We then apply this validated methodology to monitor liver metabolism during a preclinically relevant transplant model of controlled DCD (cDCD) liver grafts [38] undergoing warm ischemia, HOPE and normothermic machine perfusion, demonstrating the method's potential for real-time tissue viability assessment in transplantation applications [39,40].

## 2. Theory

**Fig. 1.**
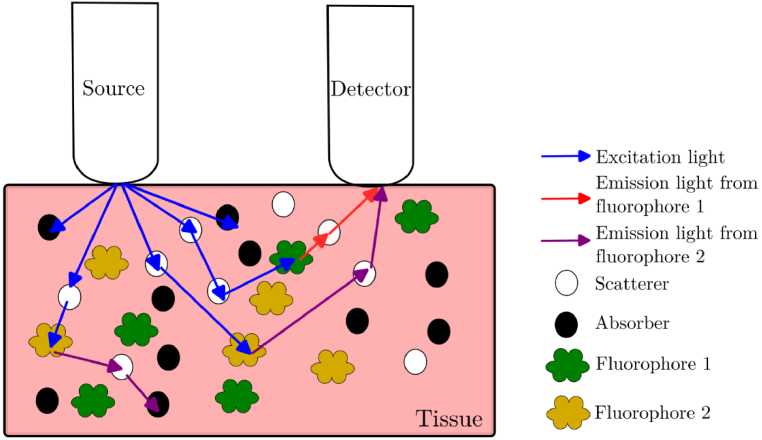
Source detector geometry used to detect fluorescence and reflectance. Excitation light goes into the tissue and can be absorbed, scattered or reemits fluorescence. This light can then reach the detector and a spectrum can be measured.

To achieve accurate fluorescence quantification in the presence of blood absorption, we employ a dual-modality fiber-optic system that combines fluorescence and diffuse reflectance spectroscopy measurements. As illustrated in Fig. 1, the system uses a source-detector geometry where excitation light is delivered through a source fiber and the resulting signals are collected by a detector fiber positioned at a fixed distance from the source [41].

The measurement principle relies on the sequential acquisition of fluorescence emission with dual excitation at 375 nm and 405 nm for our application, and diffuse reflectance from the same tissue volume. During fluorescence measurements, excitation light (blue arrows) penetrates the tissue and excites endogenous fluorophores (NADH, FAD) distributed throughout the sampling volume. The emitted fluorescence (red and purple arrows) propagates through the tissue, experiencing wavelength-dependent attenuation due to absorption by hemoglobin (black circles) and scattering by tissue components (white circles) before reaching the detector. This measured fluorescence signal is thus distorted by the tissue's optical properties.

The diffuse reflectance measurements use broadband illumination from 480 to 700 nm to correct fluorescence on the wavelength range where the most relevant fluorophores emit, and to recover the true fluorophore concentrations independently of blood content and tissue structure variations.

### 2.1. Models for fluorescence correction of the optical properties

In biological tissues, a simple model is often applied to retrieve the concentration of each fluorophore in the tissue for a given excitation wavelength:

(1) $$F_{m}(\lambda )=\underset{i}{\sum }C_{i}\cdot f_{i}(\lambda )$$

where *f_i_* represent the molar emission spectrum of the fluorophore i considered, $C_{i}$ their molar concentration and $F_{m}$ is the measured fluorescence signal. In this article we will mainly focus on retrieving the relative contribution of each fluorophore in the total fluorescence signal that we will note *c_i =_ C_i_/∑C_i_* for fluorophore *i*, between 0 and 1, leading to ∑*c_i_* =1.

This simple model does not account for the scattering and absorption properties of the tissue, leading to a possible misquantification of the concentrations. To be valid, we need to apply a precorrection that will correct measurements from those scattering and absorption effects that alters fluorescence signals. Different methods were studied to make this correction such as fluorescence attenuation correction, which gave promising results [11]. We present below two models of the literature using reflectance measurement in emission wavelength range to correct this issue. They will be used to assess the performance of our new model as compared to the state of the art.

#### 2.1.1. Kim model

The model developed by Kim et al. [42] is based on diffusion theory for light propagation in tissues. The fundamental assumption is that fluorescence photons and diffusely reflected photons follow similar path lengths through tissue, particularly when tissue absorption at the excitation wavelength is much larger than at emission wavelengths.

Kim et al. start with the relationship that the measured fluorescence *F_m_(λ)* at emission wavelength *λ*, under constant optical properties at excitation wavelength* λ_ex_* and constant fluorophore concentration, is linearly proportional to the diffuse reflectance *R* at the emission wavelength:

(2) $$F_{m}(\lambda )=S\times R(\lambda )$$

where *S* is the fraction of excitation photons launched into the tissue that are absorbed and produce fluorescence.

The term *S* can be decomposed into two components: *S = S_1_ × S_2_*, where *S_1_* is the fraction of excitation photons absorbed by the tissue and *S_2_* is the fraction of absorbed photons that are reemitted as fluorescence.

The fraction *S_1_* can be derived from the total diffuse reflectance *R_tot_* exiting the tissue at the excitation wavelength:

(3) $$S_{1}=(1-R_{tot}(\lambda_{ex}))$$

where *R_tot_* can be calculated from tissue optical properties using diffusion theory or measured directly [43].

The quantitative fluorescence *f_m_* (in units of nm^−1^ **·** cm^−1^) is defined as the product of the wavelength-dependent fluorescence quantum yield *Q_m_* and the fluorescence absorption coefficient *μ_a,fluo_* at the excitation wavelength *λ_ex_*. It represents an intrinsic property of tissue fluorophores, independent of collection geometry or tissue optical properties. The fraction *S_2_* relates the fluorescence generation to total absorption. Assuming that fluorophore absorption is negligible compared to intrinsic tissue absorption (*μ_a,fluo_*(*λ_ex_*)<< *μ_a_*(*λ_ex_*)), the total absorption can be approximated by *μ_a_*(*λ_ex_*) :

(4) $$S_{2}=\frac{Q_{m}(\lambda_{ex})\;\mu_{a,fluo}(\lambda_{ex})}{\mu_{a}(\lambda_{ex})}$$

Combining these elements, the measured (uncorrected) fluorescence at a single wavelength excitation *λ_ex_* can be expressed as:

(5) $$F_{m}(\lambda )=\frac{\left(1-R_{tot}\left(\lambda_{ex}\right)\right)\times \left(Q_{m}\left(\lambda_{ex}\right)\mu_{a,fluo}\left(\lambda_{ex}\right)\right)}{\mu_{a}\left(\lambda_{ex}\right)}\times R(\lambda )$$

Rearranging to obtain the quantitative corrected fluorescence:

(6) $$f_{m}(\lambda )=Q_{m}(\lambda_{ex})\mu_{a,fluo}(\lambda_{ex})=\frac{\mu_{a}(\lambda_{ex})}{1-R_{tot}(\lambda_{ex})}\times \frac{F_{m}(\lambda )}{R(\lambda )}$$

This equation provides a physically-based correction method that primarily addresses absorption effects using reflectance measurements. By incorporating the total diffuse reflectance at both excitation (through the term 1/(1-*R_tot_*)) and emission wavelengths (through *R*), the model corrects for the wavelength-dependent attenuation of both the excitation light reaching the fluorophores and the emitted fluorescence returning to the detector.

In the context of our work, we focus on retrieving relative fluorophore contributions from normalized fluorescence spectra rather than absolute concentrations. When normalizing spectra by their integral over wavelength (total intensity), the wavelength-independent terms in Eq. (6) simplify, leaving only the wavelength-dependent correction:

(7) $$F_{c,Kim}(\lambda )=\frac{F_{m}(\lambda )}{R(\lambda )}$$

This simplified form represents the Kim model adapted for normalized spectral analysis, where *F_c,Kim_ (λ)* is the corrected normalized fluorescence spectrum when applying Kim's method, *F_m_*(*λ*) is the measured normalized fluorescence spectrum with a total integral of 1, and *R*(*λ*) is the measured diffuse reflectance spectrum.

#### 2.1.2. Valdes model

Building on the same light transport theoretical framework, Valdes et al. recognized that the assumption of a linear relationship between fluorescence and reflectance may not fully capture the complexity of fluorescence propagation in tissue. In their work on protoporphyrin IX (PpIX) quantification during fluorescence-guided brain surgery, they observed that the main PpIX fluorescence emission peak occurs around *λ* = 635 nm. At these emission wavelengths, the balance between absorption and scattering in tissue can shift, with scattering effects becoming increasingly important. Valdes et al. therefore introduced a semi-empirical parameter *α* to better account for the influence of scattering on fluorescence propagation in the emission band. The corrected fluorescence in Valdes’ model takes the form:

(8) $$F_{c}(\lambda )=\frac{F_{m}(\lambda )}{R_{ex}\times {R_{em}}^{\alpha }}$$

where *R_ex_* is the integrated reflectance near the excitation wavelength (*λ* = 465 to 485 nm, chosen to be as close as possible to the fluorescence excitation band at *λ* = 405 nm), *R_em_* is the integrated reflectance in the emission band (*λ* = 625 to 645 nm, sampling the main PpIX fluorescence emission peak around *λ* = 635 nm), and *α* is the semi-empirical correction parameter. The parameter *α* modulates the influence of reflectance-derived optical properties on the correction. As Valdes explains in his work, this parameter allows the model to better account for scattering events that affect the relationship between measured reflectance and fluorescence attenuation. The power law formulation provides flexibility to capture deviations from the simple linear relationship assumed in Kim's model, particularly when scattering contributions become significant relative to absorption.

In Valdes' original work on protoporphyrin IX quantification during fluorescence-guided brain surgery, *α* was determined through experimental calibration on tissue-simulating phantoms with known optical properties and fluorophore concentrations. The optimal value was found to be tissue-specific and geometry-dependent. Interestingly, for brain tissue applications, they determined *α* = -0.7, a negative value that contrasts with positive values reported in other implementations [11]. In the context of our work, as with Kim's model presented previously, we focus on retrieving relative fluorophore contributions from normalized fluorescence spectra rather than absolute concentrations. When working with spectra normalized by their total intensity, the wavelength-independent terms cancel out. For the Valdes model, this means that the excitation reflectance term Rex is removed from the correction.

Furthermore, while Valdes' original approach was designed for PpIX quantification using integrated reflectance over specific wavelength bands, our objective is to correct entire fluorescence spectra for organ transplantation assessment, where multiple endogenous fluorophores (NADH, FAD) contribute across a broad spectral range. Therefore, instead of using integrated reflectance values, we apply the correction directly at each emission wavelength. The adapted Valdes model for normalized spectral correction becomes:

(9) $$F_{c,Valdes}(\lambda )=\frac{F_{m}(\lambda )\;}{R{\left(\lambda \right)}^{\alpha }}$$

here *F_c,Valdes_*(*λ*) is the corrected normalized fluorescence spectrum, *F_m_*(*λ*) is the measured normalized fluorescence spectrum, *R*(*λ*) is the measured diffuse reflectance spectrum, at each emission wavelength *λ*, and *α* is a fixed semi-empirical correction parameter.

Following Valdes’ approach, we calibrate *α* on a set of tissue simulating phantoms with known optical properties and fluorophore concentrations. Since our system employs dual-wavelength excitation at 375 nm and 405 nm, we determine one optimal *α* value for each excitation wavelength that minimizes the error between measured fluorescence and model predictions across all phantoms. This leads to two parameters, *α*_375_ and *α*_405_, which can be used to evaluate the performance of the adapted Valdes model compared to other correction approaches.

### 2.2. New model: adaptative reflectance correction

The models developed by Kim et al. and Valdes et al. provide valuable frameworks for fluorescence correction based on physical principles of light transport. However, their direct application presents challenges in organ transplantation applications, where ischemic conditions, reperfusion events, and varying degrees of tissue damage create significant spatial and temporal variations in both absorption (primarily from hemoglobin content changes) and scattering properties.

A critical limitation is the reliance on a fixed *α* parameter. Valdes' approach assumes that a single *α* value, calibrated through phantom measurements, can adequately represent all optical conditions encountered. However, tissues may transition between different optical regimes, from conditions where absorption is predominant (where Kim's linear correction with *α* = 1 could be appropriate) to conditions with important scattering (where different *α* values could be needed). A fixed parameter cannot adapt to these varying conditions.

We therefore propose an adaptive correction model that automatically determines the optimal *α* parameter for each individual measurement. This leads to the same functional form as the Valdes model:

(10) $$F_{c,new}(\lambda )=\frac{F_{m}(\lambda )}{R{\left(\lambda \right)}^{\alpha }}$$

where *F_c,new_*(*λ*) is the corrected normalized fluorescence spectrum with our new approach, *F_m_*(*λ*) is the measured normalized fluorescence spectrum, *R*(*λ*) is the measured diffuse reflectance spectrum, at each emission wavelength *λ*, and *α* is the fitted correction parameter, which is not fixed for a single excitation wavelength contrary to Valdes’ model.

In our approach, we choose to constrain *α* to the range [0, 1], where each boundary has a clear physical interpretation. A value of *α* = 1 corresponds to Kim's correction, representing the linear relationship appropriate for absorption-dominated regimes. A value of *α* = 0 indicates that no correction is necessary. Intermediate values allow the model to adapt to the particular balance between absorption and scattering effects. The optimization process involves fitting the corrected fluorescence spectrum to a linear combination of known fluorophore basis spectra while simultaneously determining the optimal *α* value. This is achieved by minimizing the residual error between the model prediction and the corrected spectrum:

(11) $$\alpha ,C_{i}=\text{arg}\;\underset{\alpha ,C_{i}}{\min}{\left|\frac{F_{m}(\lambda )}{R{\left(\lambda \right)}^{\alpha }}-\underset{i}{\sum }C_{i}\cdot f_{i}(\lambda )\right|}^{2}$$

For biological tissues, the emission spectrum of some fluorophores depends on the environment, and therefore cannot be easily measured in phantoms. Indeed, in our study the main contributions came from NADH, FAD, protein bound flavin mononucleotide (FMN), lipopigments and protoporphyrin IX with two emission peaks around 620 and 634 nm as reported in the literature [44]. Only NADH and FAD emission spectrum were measured at excitation at 375 and 405 nm in phantoms, whereas the spectrum of protein bound FMN, lipopigments and protoporphyrin were fitted based on Gaussian analytical models. To avoid overfitting, we chose to constrain them. Those constraints can be found in Table 1.

**Table 1.** Properties of the fitted fluorophores as gaussian forms (µ is the mean of the distribution and σ is the standard deviation).

Fluorophore	µ (nm)	σ (nm)
Protein bound FMN	495 ± 1	15 ± 1
Lipopigments	590 ± 1	10 ± 1
PpIX 636	637 ± 1	6.25 ± 0.75
PpIX 620	619 ± 1	8.25 ± 0.75

Those values were found empirically and correspond to literature [44,45]. They enabled to avoid overfitting. In this context, we can rewrite Eq. (11) on the following form:

(12) $$\alpha ,C_{i},\mu_{i},\sigma_{i}=\;\underset{\alpha ,C_{i},\mu_{i},\sigma_{i}}{argmin}{\left|\frac{F_{m}(\lambda )}{R{\left(\lambda \right)}^{\alpha }}-C_{NADH}f_{NADH}(\lambda )-C_{FAD}f_{FAD}(\lambda )-\underset{i}{\sum }C_{i}\cdot g_{i}(\lambda ,\mu_{i},\sigma_{i})\right|}^{2}$$

with *g_i_* being gaussian forms: $g_{i}(\lambda ,\mu_{i},\sigma_{i})=\frac{1}{\sigma_{i}\sqrt{2\pi }}\exp\left(-\frac{{\left(\lambda -\mu_{i}\right)}^{2}}{2\sigma_{i}^{2}}\right)$ .

Models adapted from Kim et al. and Valdes et al. involve the same optimization but without optimizing *α* parameter, which was fixed to 1 in Kim's model or to *α*_375_ or *α*_405_ for Valdes’ model, depending on the excitation wavelength.

To ensure physically meaningful solutions and prevent overfitting, boundary conditions were applied: 0 < *α* < 1 (as explained previously) and *C_i_* > 0 (negative fluorophore contributions are physically unrealistic). Additional constraints on the spectral parameters *µ_i_* and *σ_i_* are listed in Table 1. These constraints are essential for stabilizing the optimization, particularly given the restricted range of *α*. Optimization was performed using MATLAB's *lsqcurvefit* function with convergence criteria of either a change in residuals between consecutive iterations below 10^−6^ (criteria reached in almost every optimization presented below) or a maximum of 10^8^ iterations. To verify the robustness of the optimization, a sensitivity analysis was performed by running the algorithm with 1000 randomly selected initialization points for both phantom and porcine tissue measurements. In every case where the algorithm converged to a solution (final residuals of less than 5% of integral of *F_m_*), the optimization reached the same solution, demonstrating that the cost function exhibits a well-defined global minimum and that the results are independent of the starting point.

## 3. Material and methods

### 3.1. Optical setup for fluorescence and diffuse reflectance acquisition

**Fig. 2.**
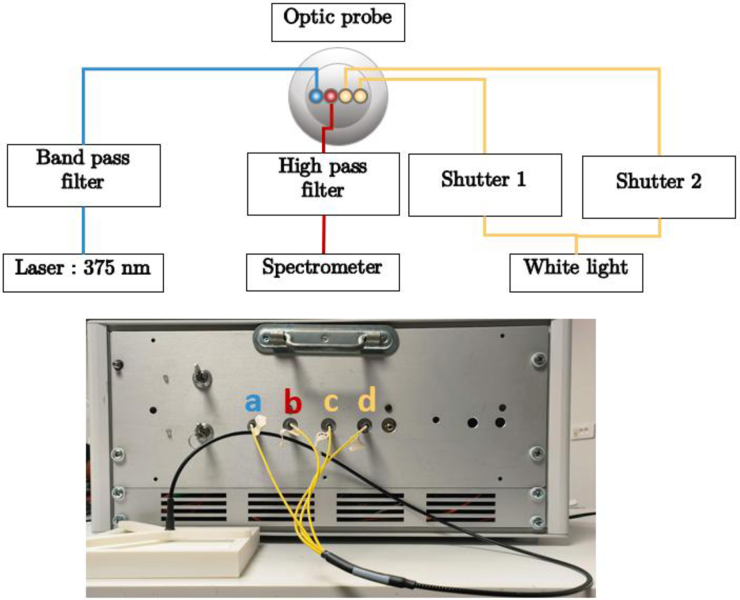
Measurement on white reflectance standard with custom experimental setup for reflectance and fluorescence measurement. One fiber of the probe is used for fluorescence excitation with both lasers (a). Two fibers emit white light for reflectance measurement (c,d) and the middle fiber is connected to the spectrometer (b).

We developed a custom dual-modality spectroscopy system combining fluorescence excitation at multiple wavelengths with broadband diffuse reflectance capabilities shown in Fig. 2. The system incorporates two laser sources operating at 405 nm (LBX-405–900-HPE-PPA, *Oxxius*) and 375 nm (LBX-375-200-HPE-PPA, *Oxxius*), wavelengths specifically selected for their ability to efficiently excite both NADH and FAD while maintaining adequate tissue penetration depth. For diffuse reflectance measurements, the system employs a broadband white light source (LS-WL-1, *Lightsource.tech*) enabling spectral characterization from 480 to 700 nm. Spectral acquisition is performed using a high-sensitivity QE-PRO spectrometer (*Ocean Optics*). A band pass filter centered at 390 nm, 40 nm full-width half-maximum (FF01-390/40-12.5, *Chroma*) was added before the probe to remove residuals coming from other modes of the lasers at higher wavelengths that could add noise to our fluorescence measurement. A high pass filter cutting at 430 nm (ET430lp, *Chroma*) was placed in front of the spectrometer to remove excitation wavelength from the laser in the fluorescence measurement. Shutter placed after the White Light (INLINE-TTL-S, *OceanInsight)* enabled to control the white light illumination.

The fiber-optic probe (custom made, *Idil*) features a four-fiber line configuration with 200 μm core diameter fibers (FG200UEP, *Thorlabs*): two dedicated to white light delivery for reflectance measurements, one for laser excitation, and one for signal collection. Two fibers are used to emit white light to use different source-detector distances depending on the application. In our case we will only use the “short” distance (fiber c on Fig. 2). This geometry therefore maintains a fixed source-detector separation of 200 μm for both fluorescence and reflectance measurements, ensuring consistent sampling volumes and measurement reproducibility.

### 3.2. Measurement protocol

Each measurement sequence begins with the probe tip positioned in gentle contact with the sample surface. The acquisition protocol follows a systematic approach with an integration time of *T* seconds:

– *N* diffuse reflectance spectra (shutter open) are first acquired with *N* background measurements (shutter closed) interspersed between each acquisition to account for ambient light variations. This gives a duration of 2*N*T seconds.
– Subsequently, fluorescence measurements are performed using a sequential protocol consisting of background acquisition (lasers off) followed by fluorescence excitation at 375 nm for *T* seconds, then another background acquisition and finally fluorescence excitation at 405 nm for another *T* seconds period, with this sequence repeated *K* times to ensure statistical reliability. This gives a duration of 4*K*T seconds.

Then, the protocol has a total duration of 2*N*T + 4*K*T seconds. All acquisitions employ a standardized *T* = 200 ms integration time and *N* = *K*= 5 at optimized excitation powers, which corresponds to a total duration of 6 seconds. Background subtraction is applied to each corresponding acquisition type, and the five repeated spectra are averaged after verification that no motion artifacts or acquisition anomalies are present. Fluorescence spectra are normalized by the input laser power to account for the linear relationship between excitation intensity and fluorescence emission at low excitation power, enabling quantitative comparison across measurements performed at different power levels. Reflectance spectra are calibrated against a certified white reflectance standard (SG 3051, *Labsphere*) using an identical acquisition protocol to compensate for source spectral variations and ensure absolute reflectance measurements.

### 3.3. Phantoms protocol

To compare the different models on phantoms with known optical properties we used different concentrations and combinations of fluorophore/chromophores. We chose to have relatively the same concentrations of TiO_2_ in the phantoms to keep a reduced scattering coefficient close to what we can find in human tissue. TiO_2_ are one of the main agents used in biomedical optics to provide scattering properties to liquid phantoms [46,47] as they are highly stable and can provide a large range of scattering properties by controlling the quantity of agent added to our solution. NADH or FAD were added to solutions of AR1, AR14, TiO_2_ at a volume and concentration chosen to obtain the desired concentrations in chromophores and fluorophores presented in Table 2.

**Table 2.** Composition of the phantoms.

N°	Fluorophore	Absorbers	AR1 *g/L*	AR14 *g/L*	TiO_2_ *mg*	FAD µ*M*	NADH µ*M*
**1**	FAD /TiO_2_	AR1	0.5		236	202	
**2**		AR1 AR14	0.5	0.46	214	201	
**3**		AR1 AR14	0.28	0.21	248	200	
**4**		AR14		0.42	224	197	
**5**	NADH/TiO_2_	AR1	0.58		229		200
**6**		AR1 AR14	0.28	0.21	244		200
**7**		AR14		0.42	250		200

**Fig. 3.**
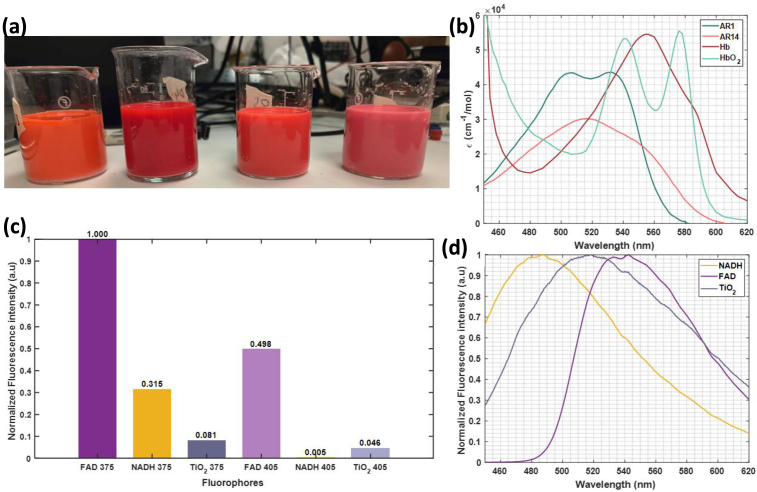
a) 4 phantoms prepared according to Table 2. b) Molar extinction coefficients of Hb, HbO_2_, AR1 and AR14. c) Emission intensities of FAD/NADH/TiO_2_ with excitation with lasers 375 and 405, normalized by the maximum intensity value of FAD emission at 375 nm excitation. d) Emission spectra of FAD/NADH/TiO_2_ at 375 nm excitation, normalized by their maximum value.

Phantoms n°2,4,5 and 6 are presented in Fig. 3(a). The measured absorption coefficient of the chromophores considered are plotted in Fig. 3(b). They were measured by diluting AR1(*Sigma*) and AR14 (*Sigma*) at a concentration of 0.025 mg/mL and 0.0245 mg/mL in deionized water.

As one can see, AR1 and AR14 spectral shape are close to the absorption spectra of Hb and HbO_2_ respectively. Therefore, by changing the ratio of concentration AR1/AR14 we can imitate StO_2_ modification. Fluorescence emission properties of FAD (*Sigma*), NADH (*Sigma*), and TiO_2_ (*Sigma*) are visible in Fig. 3(c) and (d). Their emission spectrum and intensity, regarding excitation wavelength, were measured with our setup described above in deionized water phantoms containing only the fluorophore at a concentration 200 µM, 200 µM and 50 mM respectively.

### 3.4. Porcine model protocol of cDCD liver graft

**Fig. 4.**
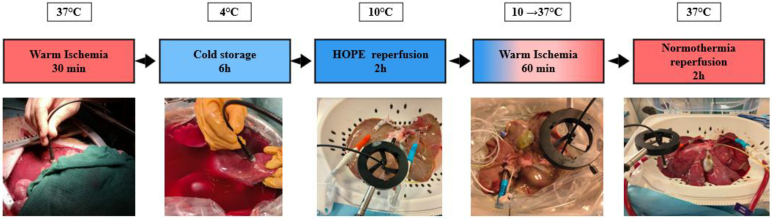
Protocol performed on porcine model and acquisitions performed during each of those steps.

The new correction method was applied to a model of cDCD liver graft. The experimental protocol was designed to simulate clinical liver preservation and transplantation conditions, allowing us to test our fluorescence measurement technique under realistic physiological stress conditions. A single porcine liver was procured following standard transplantation procedures described in Fig. 4. After 30 minutes warm ischemia representing cDCD donation [38], the liver underwent a period of 6 hours of cold storage at 4°C in preservation solution to simulate the typical preservation period during organ transport in clinical transplantation. Following cold storage, the liver was subjected to 2 hours of hypothermic oxygenated perfusion (HOPE) at 10°C. During HOPE, the liver is perfused with oxygenated preservation solution through the portal vein and hepatic artery while maintaining hypothermic conditions [48]. After HOPE treatment, a controlled period of 1 hour of warm ischemia was induced to simulate the surgical implantation phase where the organ is temporarily without perfusion. This warm ischemic period represents a critical stress condition that can significantly impact liver viability and metabolic functions. Following warm ischemia, the liver was connected to a normothermic machine perfusion system for 2 hours at 37°C to simulate the early reperfusion phase upon transplantation into recipient. During NMP, the liver was perfused with oxygenated autologous blood, allowing for metabolic reactivation and functional assessment. Optical measurements were performed at key timepoints during the whole protocol period to monitor changes in tissue metabolism and viability. Multiple measurements were taken from different areas of the liver surface to account for potential heterogeneity in tissue response. The measurement locations were carefully documented to ensure reproducibility and to correlate optical findings with biochemical markers. As the purpose of this study was to monitor the evolution of biomarkers during the perfusion protocol, we present here results spanning from HOPE through NMP. Measurements during cold storage showed no fluorescence evolution, indicating that the liver remained in a stable preservation state. The effects of warm ischemia are therefore evaluated by comparing measurements before (end of HOPE) and after (beginning of NMP) this critical transition phase. This study adheres to all applicable ethical regulations, and the experimental protocol received approval from the Institutional Animal Care and Use Committee (APAFiS #5839-2025011013096339 v2).

## 4. Results

### 4.1. Phantoms study

**Fig. 5.**
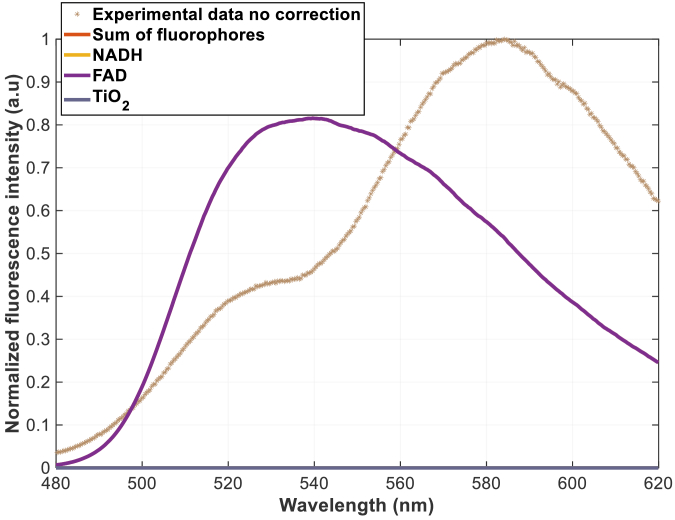
Acquired fluorescence spectrum on phantom n°3 with laser 375 nm with relative fraction of all fluorophores without correction obtained using Eq. (1). In this case only FAD is found without correction, therefore sum of fluorophores = FAD (orange and purple curves overlap).

**Fig. 6.**
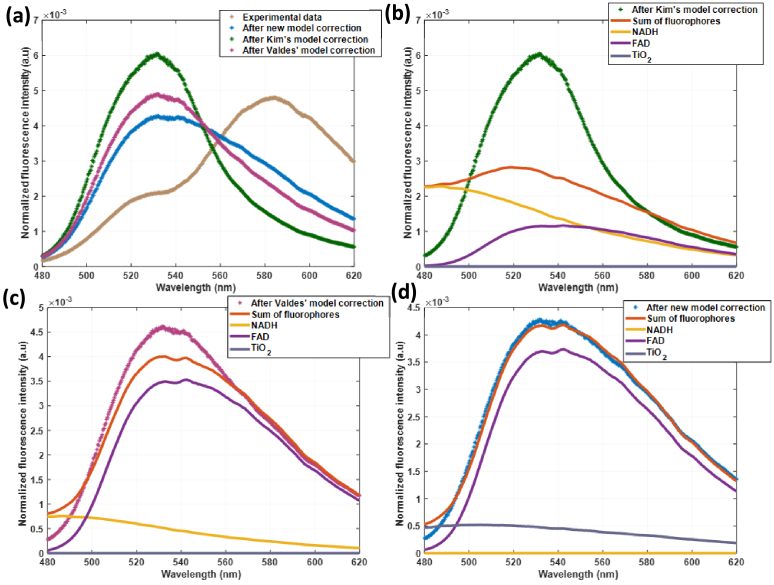
Study of phantom n°3 (FAD & TiO2 only) with laser 375 nm. a) Normalized fluorescence spectrum and normalized corrected spectrum with the 3 proposed methods, normalization of each spectrum by its integral. Corrected spectrum, sum and contribution of each fluorophore obtained with b) Kim's model, c) Valdes' model and d) new model, all spectra normalized by the corrected model studied.

The severe impact of tissue optical properties on fluorescence measurements is clearly demonstrated in Fig. 5, using phantom n°3 which contains only FAD and TiO_2_ as fluorophores, with AR1 and AR14 as absorbers. Attempting to fit fluorophore contributions directly between 480 and 620 nm without optical correction using the classical model (Eq. (1)) results in poor agreement with experimental data. The uncorrected experimental spectrum shows dramatic distortion compared to the theoretical FAD and TiO_2_ emission profiles visible in Fig. 3(d), with a pronounced absorption valley between 480–540 nm corresponding to the absorption peaks of AR1 and AR14. This distortion completely masks the true spectral shape of FAD fluorescence, making accurate quantification impossible without correction. The sum of fitted fluorophores, simply equal to FAD contribution in this case, fails to match the experimental spectrum, particularly in absorption-affected spectral regions from 500 to 600 nm. Moreover, no TiO_2_ contribution was found in this case, whereas its contribution should not be negligible here. To address these distortions, we systematically compared the three correction approaches with a fitting protocol between 480 and 620 nm, (two from literature and our new method) using the same phantom n°3 data with excitation at 375 nm. The spectral correction comparison in Fig. 6(a) reveals that each method tends to correct from the huge absorption induced by Acid reds absorption between 480 and 560 nm. However, we can observe strong differences in the obtained corrected spectrum depending on the method used. To compare the contributions obtained by each method, we show in Fig. 6(b,c,d) the fitted fluorescence spectrum for each method.

Kim's correction produces a moderately corrected spectrum that partially addresses absorption effects but exhibits overcorrection in high absorption regions, such as 500–600 nm for Acid red applications.

To perform Valdes’ correction, the parameter *α* was calibrated on all our phantoms by solving Eq. (12) with a single *α* for all phantoms at a given excitation wavelength, which led to *α*_375_ = 0.63, *α*_405_ = 0.625. Valdes’ correction with this calibrated *α* parameter produces a smoother corrected profile that better preserves overall spectral shape, with remaining discrepancies in the 480–560nm region.

Our new adaptive method yields the most faithful reproduction of the expected FAD emission profile, successfully removing absorption artifacts while maintaining spectral fidelity, especially in the peak absorption of the phantom, from 510 to 560 nm here. The fundamental limitations of the Kim correction method become apparent when examining the detailed analysis in Fig. 6(b). Overcorrection produced by Kim's model leads to systematic errors when fitted with fluorophore basis functions, incorrectly identifying substantial NADH contributions where none should exist. The FAD contribution is then underestimated, while TiO_2_ theoretical contribution is not retrieved. The poor agreement between the sum of fluorophores and corrected experimental data, resulting in high mean square error, demonstrates that this overcorrection problem is fundamental to the Kim approach.

Significant improvement is achieved with the Valdes model using calibrated parameters, as shown in Fig. 6(c). The semi-empirical *α*_375_ parameter, calibrated on our experimental data, effectively reduces the overcorrection problem, resulting in more appropriate spectral shape. However, the fixed *α*_375_ value, while optimized for the overall phantom set, remains suboptimal for this specific optical condition.

**Fig. 7.**
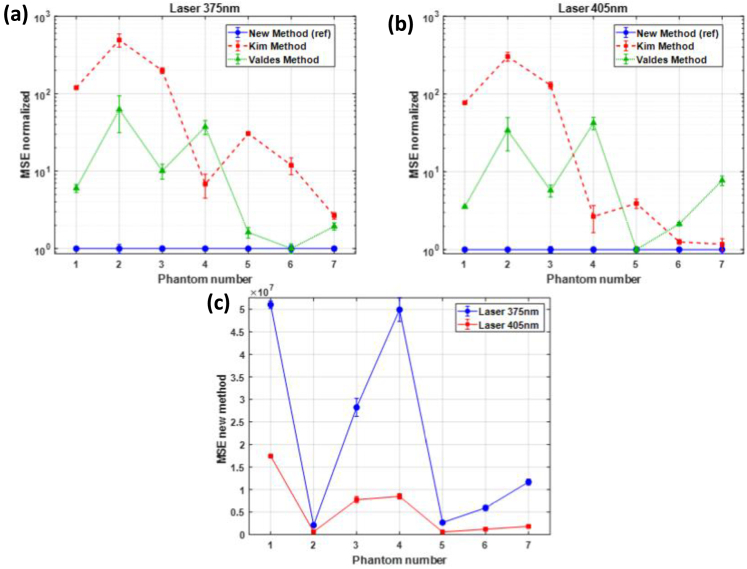
a) Mean square error normalized to new method which is labeled as ref for each phantom with excitation at a) 375 nm, b) 405 nm. MSE is calculated from Eq. (12) with each method for each phantom and is normalized by the MSE obtained with our new method. We therefore obtain a MSE normalized of 1 for our new method for each phantom. c) MSE calculated with Eq. (12) for our new method for all phantoms for both excitations.

The fluorophore fitting still incorrectly identifies a NADH contribution, though much reduced compared to the Kim method. The FAD fraction is still underestimated but closer to the theoretical value while TiO_2_ still remains inexistent. The sum of fluorophores shows better agreement with the corrected spectrum, residual fitting errors persist due to the suboptimal fixed *α*_375_ parameter. Optimal performance is achieved with our new adaptive correction method, as demonstrated in Fig. 6(d). By determining the specimen-specific *α* parameter through simultaneous fitting, the corrected spectrum perfectly reproduces the expected FAD and TiO_2_ emission profiles with negligible artifacts without adding NADH contribution as the other models did. The excellent agreement between the sum of fitted fluorophores and corrected experimental data demonstrates the effectiveness of adaptive *α* optimization for each specific measurement condition. These qualitative observations from phantom n°3 are confirmed by quantitative validation across all seven phantoms, as shown in Fig. 7. The mean square errors (MSE) values normalized to our new adaptive method for 375 nm excitation reveal dramatic performance differences (Fig. 7(a)). For FAD-containing phantoms (n°1–4), our method achieves baseline MSE values while the Valdes method shows 8–15-fold higher errors and the Kim method exhibits 100–600-fold higher errors. This massive difference demonstrates the severity of overcorrection in the Kim approach. NADH-containing phantoms (n°5–7) show similar patterns, with our method maintaining low MSE while other methods exhibit increasingly poor performance, particularly for phantom 5 where the Kim method shows >500-fold higher error.

The performance hierarchy remains consistent for 405 nm excitation, as evident in Fig. 7(b). The absolute MSE values for our method in Fig. 7(c) remain consistently low (in the order of 10^7^), compared to the intensity of fluorescence signal, across all phantoms and both excitation wavelengths, demonstrating robust performance regardless of optical conditions or fluorophore composition. This reduced MSE indicates that our model reproduces more accurately the experimental data.

**Fig. 8.**
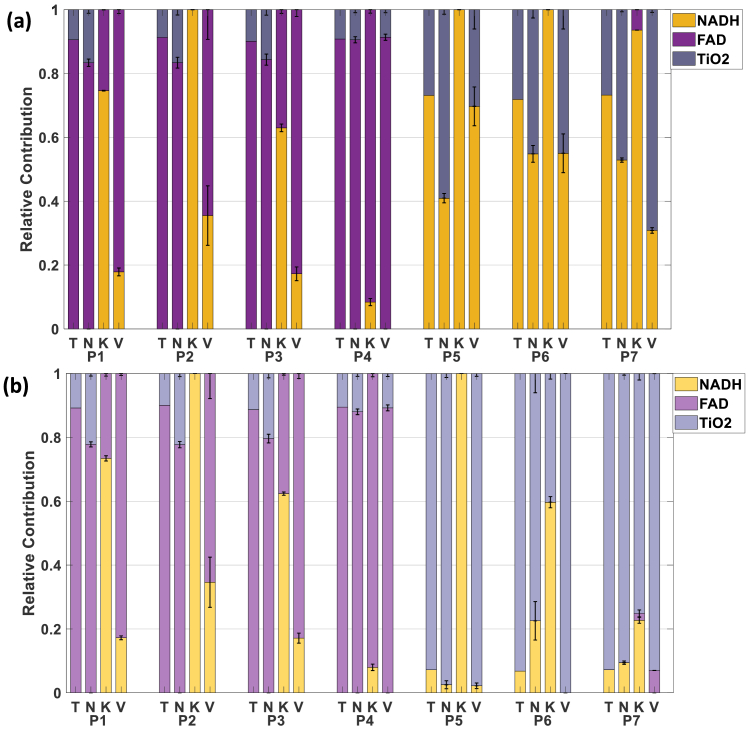
Comparison between theoretical relative contribution of fluorophores in each phantoms and contributions retrieved using the models (T: Ground Truth, N: New, K, Kim, V: Valdes) with a) 375 nm excitation b) 405 nm excitation.

The validation test involves recovery of known theoretical fluorophore fractions across all phantoms, determining whether each correction method can accurately determine true relative contributions of each fluorophore component. Our adaptive method consistently recovers theoretical fractions with minimal deviation across all seven phantoms, as shown in Fig. 8(a) for 375 nm excitation. For FAD-containing phantoms (n°1–4), recovered fractions closely match theoretical values with maximum errors below 10%, while for NADH-containing phantoms (n°5–7), the method correctly identifies important NADH contributions. In contrast, the Kim method shows systematic overestimation of NADH and underestimation of FAD across multiple phantoms, while the Valdés method shows improved but still imperfect recovery.

Similar patterns emerge for 405 nm excitation in Fig. 8(b), with our method maintaining superior accuracy. The consistent performance across both wavelengths validates the robustness of the adaptive approach, while the 405 nm results show slightly better overall fluorophore discrimination, supporting its selection for biological tissue measurements requiring balanced NADH/FAD quantification. This confirms that our model is not overfitting but can retrieve the original contribution of all the fluorophores.

The results obtained in Fig. 8 can be summarized as metrics to compare the performances of the 3 methods. Detection metrics shown in Table 3 quantify these performance differences using sensitivity, specificity adapted for fluorophore detection accuracy. Those metrics can be defined based on those definitions:

• True positive (TP): the fluorophore is present in the phantom and correctly detected by the model.
• False Positive (FP): the fluorophore is absent and incorrectly detected by the model.
• True Negative (TN): the fluorophore is absent and correctly not detected.
• False Negative (FN): the fluorophore is present and not detected by the model.

**Table 3.** Comparison of performances between the different methods.

	Sensitivity		Specificity		MAE	
	Laser 375	Laser 405	Laser 375	Laser 405	Laser 375	Laser 405
Kim	42.9	57.1	28.6	42.9	0.313	0.390
Valdes	78.6	57.1	57.1	42.9	0.127	0.084
New	**100**	**92.9**	**100**	**100**	**0.086**	**0.054**

We therefore define sensitivity, specificity as: $\text{Sensitivity}=\frac{TP}{TP+FN}$ , $\text{Specificity}=\frac{TN}{TN+FP}$ .

These metrics indicate whether the methods are able to detect the right fluorophores in our phantoms set. The mean absolute error (MAE), a quantification metric, is also calculated for all the phantoms, with both excitation wavelength for every method:

$$\mathrm{MAE}=\frac{1}{N}\sum_{i=1}^{N}\left|c_{mes,i}-c_{theo,i}\right|$$

with N= 7 phantoms * 3 fluorophores =21.

Our adaptive method achieves perfect performance (100%) for all detection metrics at 375 nm excitation and near-perfect performance (92.9–100%) at 405 nm, indicating that it is able to accurately retrieve the fluorophores present in all the phantoms. Valdes’ method shows intermediate performance (57.1–78.6%), while the Kim method exhibits poor performance (28.6–66.7%), particularly in specificity, indicating frequent false-positive fluorophore detection that would lead to incorrect diagnostics in a medical context by identifying NADH contributions instead of FAD for example.

For quantification accuracy, our method substantially outperforms existing approaches. At 375 nm excitation, the global MAE is reduced by factors of 4.0 and 1.5 compared to Kim's and Valdes' methods, respectively (MAE = 0.086 vs 0.127 and 0.313). Similar improvements are observed at 405 nm excitation, with MAE reductions of factors 7.0 and 1.5 relative to Kim's and Valdes' methods (MAE = 0.054 vs 0.084 and 0.390).

Collectively, these metrics demonstrate that our adaptive correction method not only accurately identifies which fluorophores are present but also precisely quantifies their relative contributions, addressing both qualitative and quantitative limitations of previous approaches.

To assess the repeatability of our fluorescence acquisition system, we calculated the coefficient of variation (standard deviation normalized by the mean) at 510 nm across the 5 repeated measurements performed for each phantom. This wavelength was chosen as it represents characteristic fluorescence emission for NADH, FAD and TiO2. The average coefficient of variation across all phantoms was 2.02%, indicating good measurement repeatability with minimal variations between successive acquisitions under identical conditions. Moreover, measurements were independent of the specific acquisition location on the phantom surface and insensitive to variations in probe handling and positioning, demonstrating the robustness and stability of the acquisition protocol in controlled conditions.

### 4.2. Porcine liver study

**Fig. 9.**
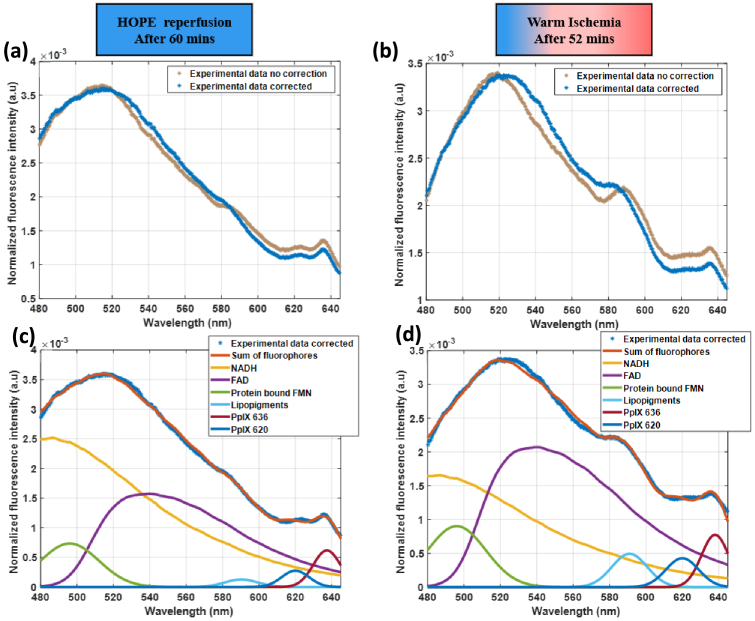
Fluorescence intensity measured with excitation at 405 nm and corrected spectra acquired during a) HOPE, b) Warm ischemia after normalization by the integral of the fluorescence spectrum. Sum and contribution of each fluorophore in the corrected spectrum after c) 60 minutes of HOPE d) 52 minutes of warm ischemia after normalization by the integral of the experimental data corrected spectrum for each spectrum.

The new validated model can be applied to the preclinical cDCD porcine model described before to assess the relative contributions of fluorophores during the different steps of the reperfusion process which show important differences in optical properties. During HOPE and warm ischemia phases, tissue optical properties remain relatively stable, resulting in minimal fluorescence distortion. Figure 9 shows representative fluorescence spectra acquired with excitation at 405 nm between 480 and 645 nm, and their corrected counterparts with our new method during these phases, demonstrating that while correction effects are modest, they remain consistently present and important for accurate fluorophore quantification.

**Fig. 10.**
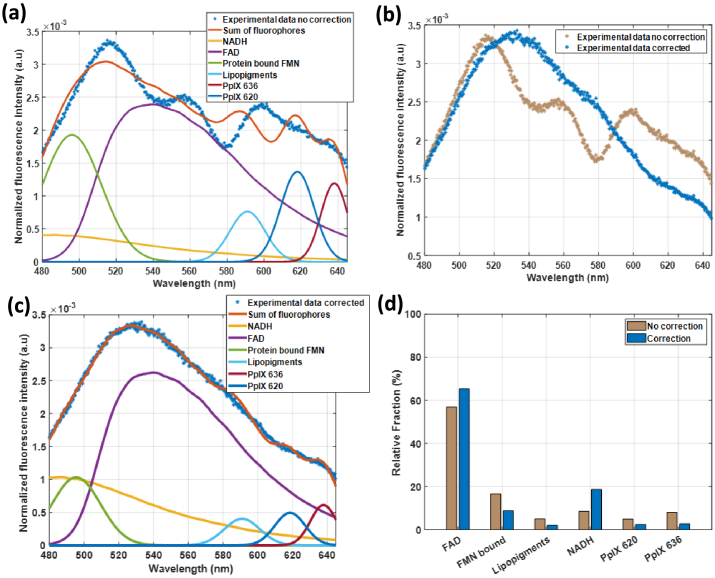
a) Fluorescence intensity measured with excitation at 405 nm, sum and fraction of each fluorophore without correction after normalization by integral of Experimental data without correction spectrum, for each spectrum. b) Fluorescence intensity with and without correction using new model after normalization by their respective integral. c) Corrected obtained spectrum, sum and fraction of each fluorophore after normalization by integral of Experimental data corrected spectrum, for each spectrum d) Comparison of relative fraction of each fluorophore with and without correction.

The correction becomes particularly critical during NMP. Figure 10 illustrates the dramatic impact of blood absorption on measured fluorescence spectra during NMP with excitation at 405 nm, with characteristic hemoglobin absorption features clearly visible between 520–600 nm. Without correction, these optical effects severely distort the measured fluorescence, leading to significant errors in fluorophore quantification and potentially misleading assessments of metabolic state as visible in Fig. 10(a). Figure 10(b) demonstrates that after correction, hemoglobin absorption features are effectively removed, enabling accurate fluorophore quantification. As shown in Fig. 10(c), the corrected spectrum exhibits excellent agreement with the sum of individual fluorophore contributions, with mean square errors values significantly reduced compared to uncorrected data. Figure 10(d) illustrates the impact of optical properties correction on individual fluorophore quantification. The comparison between corrected and uncorrected relative fractions clearly demonstrates substantial differences in the derived relative fraction of each fluorophore. Without correction, the distorting effects of tissue optical properties lead to errors in fluorophore quantification, potentially misrepresenting the true metabolic state of the tissue during NMP.

#### 4.2.1. Wavelength-specific considerations for metabolic monitoring

**Fig. 11.**
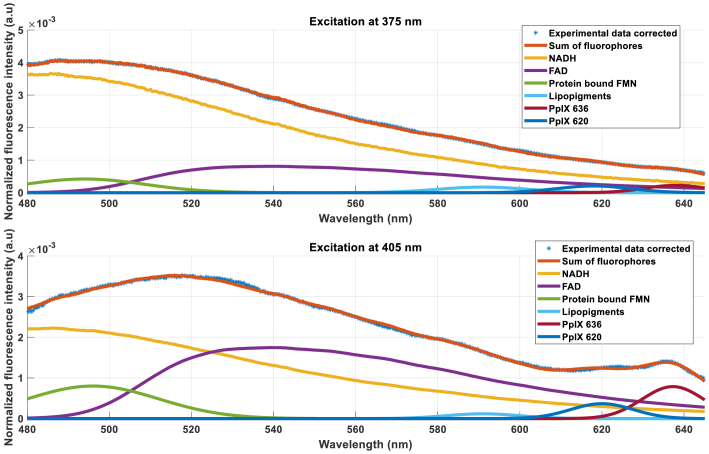
Fluorescence intensity measured with both lasers after correction, sum and fraction of each fluorophore for during normothermia reperfusion after normalization by integral of Experimental data corrected spectrum, for each spectrum.

Our study focuses on monitoring the evolution of relative fractions of each fluorophore during the preservation protocol. As observed in Fig. 11, fluorescence at 375 nm excitation is composed almost entirely of NADH contributions, with the relative fraction of NADH approaching 100%. This overwhelming dominance of NADH signal provides limited information for relative fraction analysis, as it essentially eliminates the ability to monitor changes in the balance between different fluorophores.

This observation is consistent with previous literature reports [49] demonstrating preferential NADH excitation at shorter wavelengths. While this wavelength specificity could be valuable for other applications requiring precise NADH quantification, such as determining absolute NADH concentrations rather than relative fraction, it is of limited utility for our current study focused on metabolic balance assessment.

In contrast, 405 nm excitation provides a more balanced fluorescence response from both NADH and FAD, enabling meaningful calculation of relative fractions and the optical redox ratio defined as *c_FAD_*/(*c_FAD_+c_NADH_*). This balanced response is essential for monitoring shifts in cellular metabolic state during the preservation and reperfusion protocol.

Consequently, subsequent analyses focus primarily on 405 nm excitation data, which provides the best sensitivity for tracking relative changes in fluorophore contributions, the primary objective of this metabolic monitoring study.

#### 4.2.2. Monitoring of the liver during reperfusion protocol

**Fig. 12.**
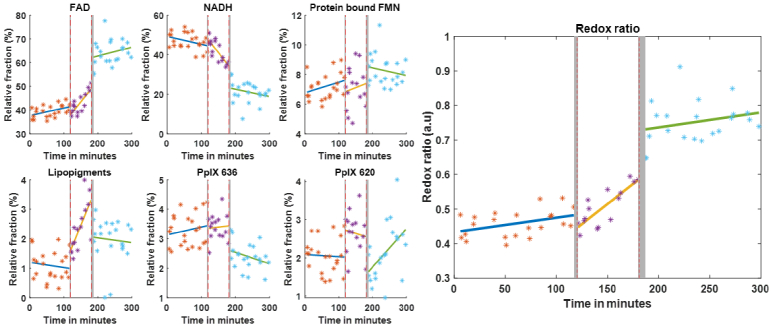
Evolution of biomarkers during HOPE/Warm ischemia/NMP according to the protocol described in Fig. 4. Evolution of the relative fraction of each fluorophore and redox ratio during a full protocol and linear fit during each step. Each star represents a measurement, orange corresponds to HOPE, purple to warm ischemia and blue to NMP. Grey areas correspond to timing where measurement could not be performed during the transitions from HOPE to warm ischemia and warm ischemia to NMP.

Figure 12 provides comprehensive monitoring of tissue metabolites throughout the entire HOPE/warm ischemia/normothermic perfusion protocol, demonstrating the clinical potential of our approach for real-time tissue viability assessment.

The evolution of individual fluorophore contributions throughout the protocol (Fig. 12(a)) reveals critical metabolic insights. The plotted values represent the relative fraction *c_i_* of each fluorophore *i*, where *c_i_* is the contribution coefficient described in the section 2.1.

During the HOPE phase, FAD relative fraction exhibits values ranging from approximately 30% to 55%, while NADH ranges from approximately 30% to 45%. A linear fit of each phase reveals a gradual increasing trend for FAD, accompanied by a corresponding decrease in NADH fraction. The protein-bound FMN, lipopigments, and PpIX fractions remain relatively stable at low levels (below 15%) throughout this phase, showing minimal variation. The redox ratio shows an almost constant value of around 0.4, with the linear fit revealing a really small positive slope.

During the warm ischemia period, the FAD fraction shows fluctuations between 30% and 60%, while NADH varies between 25% and 55%. The minor fluorophores (protein-bound FMN and 2 forms of PpIX) continue to show minimal contributions, remaining below 15%. Interestingly, lipopigments fraction increases from 1 to 3% with a visible modification of the fluorescence spectrum marked by the peak visible around 590 nm on Fig. 9(d), due to lipopigments contribution. Here, the redox ratio shows marked increase compared to the preceding step with an increase from 0.4 to 0.6.

During normothermic reperfusion, a clear linear trend emerges. The FAD fraction progressively increases from approximately 40% at the start of NMP to values exceeding 60% by the end of the 2-hour perfusion period. Conversely, NADH fraction decreases from around 45–50% to approximately 30%. The linear fit during NMP shows a consistent positive slope for FAD and negative slope for NADH, with measurement points closely following these trends. The other fluorophores (protein-bound FMN, lipopigments, PpIX) maintain stable low fractions throughout NMP, consistently below 10–15%. Especially, lipopigments fraction decreases to a value of around 2%, consistent during the whole NMP which is slightly higher than the fraction observed during HOPE of around 1%. Redox ratio comes back to a really low increase from 0.72 to 0.78, with an increase per minute similar to what is observed during HOPE.

## 5. Discussion

This study presents a comprehensive validation of a novel optical properties correction methodology for quantitative fluorescence measurements in complex biological tissues using diffuse reflectance. The validation demonstrates the robustness of our approach through a rigorous progression from controlled phantom studies to a preclinically relevant porcine liver reperfusion model. While this study does not aim to provide comprehensive biological insights into liver physiology from a single organ model, it demonstrates a concrete biomedical application of our correction methodology and shows that it can effectively extract relevant biomarkers by efficiently correcting for blood absorption in complex biological media, extending beyond phantom validation.

### 5.1. Improved fluorophore quantification demonstrated through phantom validation

Our methodology significantly surpasses state of the art performance in fluorophore identification and quantification, a considerably more challenging problem due to overlapping emission spectra and the confounding effects of tissue absorption/scattering on fluorescence signals. The phantom studies provide rigorous quantitative validation of fluorophore recovery accuracy through direct comparison with established correction methods.

The comparative analysis presented in Fig. 7 and Fig. 8 reveals dramatic performance differences between correction approaches. This massive difference demonstrates that fixed correction parameters, as used in traditional methods, systematically fail to account for the specimen-specific relationship between optical properties and fluorescence distortion.

Most critically, our method achieves accurate recovery of known theoretical fluorophore fractions across all phantom compositions. In contrast, Kim and Valdes methods show systematic bias, frequently misidentifying fluorophore contributions and for example, detecting significant NADH contributions in FAD-only phantoms or vice versa, giving a maximum specificity of 28.6% and 57.1% respectively, and a sensitivity of 78.6% and 57.1% respectively, while our method reaches 100% for both metrics and a decrease by a factor of a least 1.5 for the MAE, indicating a better result in quantification of relative fraction of fluorophores. Such false-positive fluorophore detection would lead to incorrect metabolic assessments in clinics. The improved diagnostic performance metrics achieved by our adaptive method versus traditional approaches confirm this critical advantage.

### 5.2. Residual errors and method limitations

Despite the significant improvements achieved by our correction approach, some residual errors persist in fluorophore quantification across different phantom compositions with differences that can reach a 20% difference for phantom n°5 in NADH and TiO_2_ quantification for example (Fig. 7(a)). While these errors are in average much smaller than those observed with uncorrected measurements or existing correction methods, they highlight the fundamental limitations of spectral unmixing in complex optical environments. These residual errors likely stem from the empirical nature of the *α* parameter used in our optical properties’ correction model. This empirical correction, while effective across our tested conditions, may not capture all the nuances of light-tissue interactions in every biological scenario and could potentially be refined through more sophisticated optical modeling approaches.

### 5.3. Importance of fluorophore selection and overfitting prevention

One crucial point to use this correction method is the absolute necessity of proper fluorophore selection to prevent overfitting in spectral unmixing. While our method provides excellent fitting capabilities, the risk of overfitting becomes significant when too many fluorophores are included in the model compared to what is actually present in the tissue. The key challenge is not only having precise knowledge of reference spectra, but also ensuring that only the fluorophores that are genuinely present in the biological system are included in the fitting model. The actual contribution of each of the considered fluorophore should be investigated before being added as a reference fluorophore in the fitting process.

During our phantom experiments, we observed that including unnecessary fluorophores in the unmixing model can lead to spurious contributions being assigned to fluorophores that are not actually present in the sample, especially with Kim and Valdes models as highlighted by Table 3. However, the new method proposed is also affected by this issue which can occur if we add more possible fluorophores when fitting Eq. (12).

In the porcine liver application, we carefully selected a fluorophore library consisting of NADH, FAD, protein-bound FMN, lipopigments, and protoporphyrin IX (PpIX) based on established literature regarding endogenous liver fluorophores. This selective approach prevents the algorithm from fitting noise or optical artifacts as fluorescence from biologically irrelevant compounds. However, the choice of fluorophore library remains a critical user decision that requires domain knowledge and careful validation for each new application or tissue type.

### 5.4. Repeatability assessment

While measurement repeatability was thoroughly evaluated in phantoms with an average coefficient of variation of 2.02%, a comprehensive assessment in biological tissue remains to be conducted. Such an evaluation would require measurements at multiple liver sites with repeated acquisitions at identical locations. In our porcine liver study, we observed progressive fluorescence intensity decrease between successive acquisitions, likely due to photobleaching. Despite this temporal decay, the coefficient of variation from normalized fluorescence intensities across acquisitions remained comparable to phantom studies, suggesting preserved spectral shape. Regarding probe-tissue contact pressure variations, which could alter local blood content and optical properties, our correction methodology should inherently compensate for such effects. Blood content changes primarily affect tissue absorption, which is directly captured by reflectance measurements and incorporated into our new correction algorithm. The method should therefore be robust to moderate pressure induced variations. A dedicated repeatability study in biological tissue would be valuable to formally quantify these effects and confirm the methodology's robustness. Such a protocol could include systematic measurements at multiple predefined locations with controlled probe positioning and deliberate pressure variations.

### 5.5. Metabolic monitoring during liver preservation

Having established the robustness of our correction methodology, we now examine how the fluorophore evolution patterns during a transplant protocol on porcine liver, documented in the results section, relate to expected metabolic behavior. The gradual redox ratio increase observed during HOPE aligns with expectations for hypothermic oxygenated perfusion, where reduced metabolic rate at 10°C combined with oxygen availability should make mitochondrial respiratory chains work again in an aerobic behavior, inducing a decrease in NADH concentration, an increase in FAD concentration and therefore an increase of the redox ratio [10,50]. The redox ratio increase during warm ischemia likely reflects progressive metabolic dysregulation as one can expect a decrease in redox ratio due to hypoxic stress and anaerobic glycolysis [51,52]. The lipopigments accumulation observed during warm ischemia could also serve as an indicator of cellular damage or stress applied to cells, as these compounds may be released or become more detectable when cells undergo membrane degradation and death [53,54]. However, results should be taken with caution. Our experimental design transitions directly from oxygenated HOPE to warm ischemia, creating a complex metabolic scenario where the liver enters ischemia in an oxidized state. Upon NMP initiation, the sustained redox ratio increase is consistent with restoration of oxidative phosphorylation as oxygenated blood perfusion reestablishes mitochondrial electron transport chain function [34,50]. These interpretations represent correlations with expected behavior rather than definitive mechanistic conclusions. This single-organ experiment serves primarily as a proof-of-concept demonstration that our correction methodology can track metabolically relevant fluorescence changes throughout complex preservation protocols. The observed patterns align with fundamental principles of cellular bioenergetics, but establishing quantitative relationships between optical measurements and specific metabolic parameters requires systematic studies with controlled interventions and correlation with biochemical assays (ATP levels, lactate production, oxygen consumption rates). The clinical utility lies in providing real-time, continuous metabolic monitoring to detect deviations from expected recovery patterns, organs that fail to show appropriate redox ratio evolution would be flagged as potentially compromised, while those demonstrating robust patterns would provide confidence in graft viability.

## 6. Conclusion

### 6.1. Future improvements through dual-wavelength excitation

Looking toward future improvements, the availability of dual-wavelength excitation presents an exciting opportunity for enhanced fluorophore discrimination and quantification accuracy. Our results demonstrate that 375 nm excitation provides enhanced sensitivity for NADH detection, while 405 nm excitation offers better overall performance across multiple fluorophores. A dual-laser approach could leverage the complementary information from both wavelengths to improve spectral unmixing, particularly in distinguishing NADH from FAD contributions. The preferential excitation of NADH at 375 nm could serve as a constraint in the spectral fitting algorithm, potentially reducing cross-talk between these metabolically important fluorophores and providing more robust quantification of the redox ratio. This dual-excitation method could also be used to extract not only relative fraction of fluorophores but real concentration, adding more information for clinical interpretation.

### 6.2. Clinical translation and transplantation applications

The clinical potential of this methodology extends far beyond the controlled phantom environment, particularly in the context of organ transplantation where objective viability assessment remains a critical need. The ability to quantitatively monitor tissue metabolism through NADH and FAD fluorescence could provide real-time feedback on organ condition during preservation, perfusion, and transplantation procedures. Future clinical validation studies should focus on correlating our optical measurements with established clinical biomarkers such as aminotransferase levels (AST, ALT), which serve as gold standard indicators of hepatocellular damage and organ viability. Such correlations would establish the clinical relevance of optically-derived redox ratios and potentially enable earlier detection of organ dysfunction than current biochemical markers allow. The integration of this optical approach into clinical transplantation protocols could revolutionize organ assessment, moving from subjective visual evaluation and delayed laboratory results toward real-time, quantitative metabolic monitoring.

We have successfully developed and validated a robust optical properties correction methodology that enables accurate quantitative fluorescence measurements in complex tissue-like environments through a correction using measured diffuse reflectance. Our approach significantly outperforms existing correction methods, achieving superior accuracy in fluorophore quantification across diverse optical conditions. The method's ability to reliably extract metabolic information through NADH and FAD measurements, while correcting for confounding tissue optical properties, represents a significant advancement toward objective, real-time tissue viability assessment.

The phantom validation studies demonstrate the method's robustness and establish its potential for clinical translation, particularly in applications requiring precise metabolic monitoring such as organ transplantation. Future work will focus on clinical validation studies and the implementation of dual-wavelength excitation strategies to further enhance quantification accuracy. This technology promises to transform tissue assessment from subjective evaluation to objective, quantitative metabolic monitoring, ultimately improving clinical outcomes through more informed decision-making based on real-time tissue viability data.

## Data Availability

Data underlying the results presented in this paper are not publicly available at this time but may be obtained from the authors upon reasonable request.
